# Identification of chemoresistance‐related mRNAs based on gemcitabine‐resistant pancreatic cancer cell lines

**DOI:** 10.1002/cam4.2764

**Published:** 2019-12-11

**Authors:** Jiarong Zhou, Linshi Zhang, Huilin Zheng, Wenhao Ge, Yu Huang, Yingcai Yan, Xiaohu Zhou, Wei Zhu, Yang Kong, Yuan Ding, Weilin Wang

**Affiliations:** ^1^ Department of Hepatobiliary and Pancreatic Surgery The Second Affiliated Hospital Zhejiang University School of Medicine Hangzhou Zhejiang China; ^2^ Key Laboratory of Precision Diagnosis and Treatment for Hepatobiliary and Pancreatic Tumor of Zhejiang Province Hangzhou Zhejiang China; ^3^ School of Biological & Chemical Engineering Zhejiang University of Science and Technology Hangzhou Zhejiang China; ^4^ Research Center of Diagnosis and Treatment Technology for Hepatocellular Carcinoma of Zhejiang Province Hangzhou Zhejiang China; ^5^ Clinical Medicine Innovation Center of Precision Diagnosis and Treatment for Hepatobiliary and Pancreatic Diseases of Zhejiang University Hangzhou Zhejiang China; ^6^ Clinical Research Center of Hepatobiliary and Pancreatic Diseases of Zhejiang Province Hangzhou Zhejiang China

**Keywords:** GEM‐resistant cell lines, pancreatic cancer, RRM1, STIM1, TRIM21

## Abstract

Gemcitabine (GEM) alone and GEM‐based chemotherapy are the preferred regimens for treating advanced unresectable and metastatic pancreatic cancer (PC). However, these treatments have limited efficacy due to acquired resistance of cancer cells to chemotherapy, the mechanisms of which are not fully understood. In this study, we established two stable multidrug‐resistant cell lines, BxPC‐3‐GR and CFPAC‐1‐GR, from their corresponding parental cells through exposure to GEM following a stepwise incremental dosing strategy. The GEM IC_50_ values of BxPC‐3‐GR and CFPAC‐1‐GR increased 112‐fold and 210‐fold, respectively, compared to parental cell lines. In vitro and in vivo experiments confirmed that both GEM‐resistant cell subgroups declined in proliferative capacity, but were more resistant to GEM. Unlike CFPAC‐1‐GR, BxPC‐3‐GR exhibited enhanced migratory and invasive properties compared with BxPC‐3 in vitro. We also compared differentially expressed mRNA profiles between parental and GEM‐resistant cells using transcriptome sequencing. RRM1, STIM1, and TRIM21 were significantly upregulated in both GEM‐resistant cell lines and confirmed to be associated with the degree of GEM resistance by quantitative reverse‐transcription polymerase chain reaction and western blot analysis. These three genes were more highly expressed in PC tissues and potentially regarded as prognostic biomarkers through database mining. Thus, our findings provide chemo‐resistant cell models to better understand the underlying mechanisms of chemoresistance, and to explore potential biomarkers for GEM response in PC patients.

## Graphical abstract

Our findings provide chemo‐resistant cell models to better understand the underlying mechanisms of chemoresistance and to explore potential biomarkers for gemcitabine response in pancreatic cancer patients

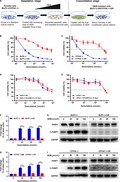

## 1 INTRODUCTION

Pancreatic cancer (PC) is the fourth highest cause of cancer‐related deaths in the United States, with 56 770 new cases diagnosed yearly.^1^ Due to its aggression, PC is characterized by high mortality and poor prognosis. Surgical resection is considered the most effective PC treatment strategy; however, due to a lack of obvious symptoms and effective tumor biomarkers, relatively few diagnosed patients can undergo initial resection before progression to the advanced stage.^2^ Therefore, chemotherapy has become increasingly important for the treatment of locally advanced, unresectable, metastatic PC patients.^3^ Gemcitabine (GEM) alone and GEM‐based chemotherapy have been accepted as standard treatments for PC; however, intrinsic or acquired resistance of cancer cells to GEM leads to disappointing outcomes in PC patients.^4^, ^5^

To date, several important molecular targets and pathways related to GEM resistance have been explained in detail. These mechanisms can be summarized by the following factors: regulation of drug transport and metabolism, DNA damage repair pathway activation, apoptosis signaling pathway regulation, pro‐survival signaling pathway activation, and epithelial‐mesenchymal transition.^6^, ^7^, ^8^, ^9^ The tumor microenvironment is an important factor for chemoresistance in multiple types of tumors including PC, allowing cancer cells to evade apoptosis by releasing specific cytokines and growth factors.^10^, ^11^, ^12^ However, the exact mechanism of action is not fully understood and irreversible therapeutic resistance remains a challenge for patients receiving chemotherapy. Therefore, an extensive study of the mechanisms underlying GEM resistance in PC may help to identify potential biomarkers for the prediction of chemoresistance and prognosis of patients receiving GEM‐based chemotherapy.

In the present study, we established two stable GEM‐resistant cell lines from corresponding parental cell lines that are relatively sensitive to GEM by continuous exposure to increasing GEM concentrations. Through a series of comparative experiments and transcriptome sequencing, we explored differential biological and molecular characterization between GEM‐resistant and parental cell groups. We also investigated the correlation of potential targets to the degree of GEM resistance, to identify biomarkers for response to GEM in PC patients.

## 2 MATERIALS AND METHODS

### 2.1 Cell lines and drugs

Human pancreatic ductal epithelial and PC (AsPC‐1, BxPC‐3, CFAPC‐1, PANC‐1, and MIA PaCa2) cell lines were obtained from our institute. Human pancreatic ductal epithelial, PANC‐1, and MIA PaCa2 were cultured in DMEM, AsPC‐1, and BxPC‐3 in RPMI 1640, and CFPAC‐1 in IMDM. All cell lines were cultured in medium supplemented with 10% fetal bovine serum (FBS), 100 µg/mL streptomycin, and 100 IU/mL penicillin and grown in a humidified incubator with 5% CO_2_ at 37°C. GEM (cat. no. S1714), 5‐flourouracil (cat. no. S1209), capecitabine (cat. no. S1156), oxaliplatin (cat. no. S1224), cisplatin (cat. no. S1166), docetaxel (cat. no. S1148), and irinotecan (cat. no. S2217) were obtained from Selleck Chemicals. All drugs were dissolved in their respective optimal solvents and stored at −20°C.

### 2.2 Establishment of GEM‐resistant cell lines

We established GEM‐resistant cell lines following a previously described method.^13^ Briefly, the method comprises two steps. In the adaptation stage, BxPC‐3 cells were treated with GEM for 48 hours by a stepwise increase in drug concentration from 20 to 500 nmol/L (20, 50, 100, 250, and 500 nmol/L), whereas CFPAC‐1 cells were exposed to GEM ranging from 10 to 200 nmol/L (10, 20, 50, 100, and 200 nmol/L). After each dose‐induced step, we discarded apoptotic cells and amplified surviving cells in GEM‐free culture medium; this step was repeated three times. Cells were then exposed to the next increment of GEM. In the consolidation stage, previously selected cells were treated with different final concentrations of GEM (400 nmol/L for CFPAC‐1 and 1000 nmol/L for BxPC‐3), until they grew normally in conditioned medium.

### 2.3 Cell morphology and cell proliferation in vitro

Cells (2 × 10^5^) were seeded into six‐well plates for 24 hours and then photographed using an inverted microscope. For cell proliferation analysis, cells (2 × 10^3^) were seeded into 96‐well plates, and the growth curve was evaluated using the Cell Counting Kit‐8 (Dojindo Molecular Technologies, Inc) for 96 hours following the manufacturer's protocol.

### 2.4 In vitro migration and invasion assays

To test motility, 24‐well transwell chambers (Corning) were used in the absence or presence of Matrigel (BD Biosciences) to assess migration or invasion, respectively. Briefly, 600 µL medium supplemented with 10% FBS was added to lower parts of the chambers, whereas the upper chambers were filled with 200 µL serum‐free medium containing 8 × 10^4^ cells. Migration cells were then fixed, stained, and photographed after 24 hours of incubation. For cell invasion analysis, the same processes were followed except that each chamber bottom was pre‐coated with 20 µg Matrigel and incubated for 48 hours.

### 2.5 3‐(4,5‐Dimethylthiazol‐2‐yl)‐2,5‐diphenyltetrazolium bromide assays

Cells (4 × 10^3^/well) were seeded into 96‐well plates for one night of incubation. Adherent cells were treated with different drug concentrations for 48 hours. Each drug concentration was repeated six times. After 48 hours of incubation, 15 µL 0.5% 3‐(4,5‐dimethylthiazol‐2‐yl)‐2,5‐diphenyltetrazolium bromide (MTT) solution (Solarbio, Inc) was added to each well for another 3 hours of incubation. And then, 200 µL of dimethyl sulfoxide (Solarbio, Inc) was substituted for medium containing MTT to dissolve formazan crystals by shaking the plate well for 5 minutes, and absorbance was detected at 560 nm using an iMark microplate absorbance reader (Bio‐Rad).

### 2.6 Flow cytometry analysis

Cell apoptosis and cell cycle were detected using Digital BD LSR II flow cytometry (BD Biosciences). For cell apoptosis analysis, cells at 40% of density were treated with different drug concentrations for 48 hours and then detected using Annexin V, FITC Apoptosis Detection Kit (Dojindo Molecular Technologies, Inc) following the manufacturer's protocol. For cell cycle progression assessment, 1 × 10^5^ cells were seeded into six‐well plates for 48 hours. Cells were then harvested and stored in 75% ethanol for 24 hours at −20°C and then mixed with DNA staining solution (Multi Science) in the dark for 30 minutes to detect cell cycle distribution.

### 2.7 Animal experiments

All animal programs were approved by the Zhejiang Medical Experimental Animal Care Commission and executed in accordance with institutional ethical guidance. As described previously,^14^ xenograft tumors were generated in nude mice by subcutaneous injection of 3 × 10^6^ parental and GEM‐resistant cells. Once the average tumor volumes reached 50 mm^3^, nude mice inoculated with each cell type were randomly divided into two groups with different treatments: (a) control vehicle (saline) and (b) 100 mg/kg GEM. Mice were injected intraperitoneally with GEM or saline every 4 days for a total of six consecutive injections, and tumor volume and body weight were measured every 4 days. Tumor volume was calculated using the formula: *V* = *L* × *W* ^2^/2, where *V* is the volume and *L* and *W* are the longest and shortest tumor diameters, respectively.

### 2.8 Hematoxylin‐eosin and immunohistochemistry

When mice were terminated, tumor samples were removed, fixed in 4% polyformaldehyde solution, and then embedded in paraffin. Tumor tissue sections were stained with hematoxylin and eosin (H & E) to observe morphology. Primary antibody against proliferating cell nuclear antigen (PCNA; cat. no. 10205‐2‐AP; Proteintech) was used to assess cell proliferation at 1:500 dilution.

### 2.9 Western blot analysis

Standard protocols for western blot were performed as previously described.^15^ Antibody against glyceraldehyde 3‐phosphate dehydrogenase (GAPDH) (cat. no. 60004‐4‐Ig; Proteintech) was used as a loading control. Various primary antibodies against PARP1 (cat. no. 13371‐1‐AP; Proteintech), cleaved PARP1 (cat. no. ab32064; Abcam), RRM1 (cat. no. ab137114; Abcam), STIM1 (cat. no. ab108994; Abcam), and TRIM21 (cat. no. ab207728; Abcam) were used to detect protein expression.

### 2.10 RNA extraction and quantitative reverse‐transcription polymerase chain reaction

Total cellular RNA was extracted and reverse‐transcribed (RT) to cDNA using TRIzol reagent (Invitrogen) and PrimeScript RT reagent kit (TaKaRa Biotechnology), respectively, according to the manufacturer's instructions. qPCR was performed using FastStart Universal SYBR Green Master (Rox) (Roche Applied Science) on the ABI 7500‐Fast Real‐Time PCR System (Applied Biosystems) following the manufacturer's protocol. Relative mRNA expression was normalized to GAPDH and calculated using the 2^–ΔΔ^ *^C^* ^q^ method.^16^ Reactions were performed in triplicate. The primer sequences were as follows: RRM1, forward, 5′‐GCCGCCAAGAACGAGTCAT‐3′ and reverse, 5′‐AGCAGCCAAAGTATCTAGTTCCA‐3′; STIM1, forward, 5′‐AGTTTTGCCGAATTGACAAGC‐3′ and reverse, 5′‐GTGGATGTTACGGACTGCCT‐3′; TRIM21, forward, 5′‐TCAGAGCTAGATCGAAGGTGC‐3′ and reverse, 5′‐ACTCACTCCTTTCCAGGACAAT‐3′; GAPDH, forward, 5′‐GGAGCGAGATCCCTCCAAAAT‐3′ and reverse, 5′‐GGCTGTTGTCATACTTCTCATGG‐3′.

### 2.11 Transcriptome sequencing analysis

Total RNA was collected from GEM‐resistant and parental cells. Briefly, cells at 80% of confluence were lysed using TRIzol reagent (Invitrogen) and scraped on ice. The miRNeasy Mini Kit (50) (cat. no. 217004; Qiagen) was used to extract and purify cellular RNA. The Agilent 2100 Bioanalyzer (Agilent Technologies) and Nanodrop one/qubit 3.0 (Thermo Fisher Scientific) were used to qualify and quantify the sample libraries during the quality control steps. Finally, all libraries were sequenced using the Illumina Novaseq 6000 sequencer (Illumina). These sequence data have been submitted to the Gene Expression Omnibus database under accession number http://www.ncbi.nlm.nih.gov/geo/query/acc.cgi?acc=GSE140077. For mRNA analysis, human GRCh 38.91 was used as the reference genome. Differentially expressed mRNAs between GEM‐resistant and parental cells were determined by the DESeq2 software package; significance was determined by the following criteria: false discovery rate <0.05 and log2 fold change magnitude (|Log2FC|)> 1. The KOBAS 3.0 database was used for Gene ontology (GO) and Kyoto Encyclopedia of Genes and Genomes (KEGG) pathway enrichment analyses.^17^ Gene ontology terms and pathways were considered significantly enriched with a *Q* value <0.05.

### 2.12 Database mining

Gene Expression Profiling Interactive Analysis (GEPIA) is an online tool for analyzing RNA sequencing expression data from TCGA and GTEx projects.^18^ The database was used to analyze differences in RRM1, STIM1, and TRIM21 expression between PC and corresponding normal tissues, and to assess correlations in gene expression. The Kaplan‐Meier plotter (KM plotter) database was used to analyze the prognostic values of the three targeted genes in PC.^19^ A level of *P* < .05 was considered as significance.

### 2.13 Statistical analyses

Data are expressed as means ± SD. All statistical analyses were performed on GraphPad Prism version 5.0 software (GraphPad Software Inc) using Student's *t* test. A level of *P* < .05 was considered as significance.

## 3 RESULTS

### 3.1 Establishment and characterization of irreversible GEM resistance in PC cell lines

**Figure 1:**
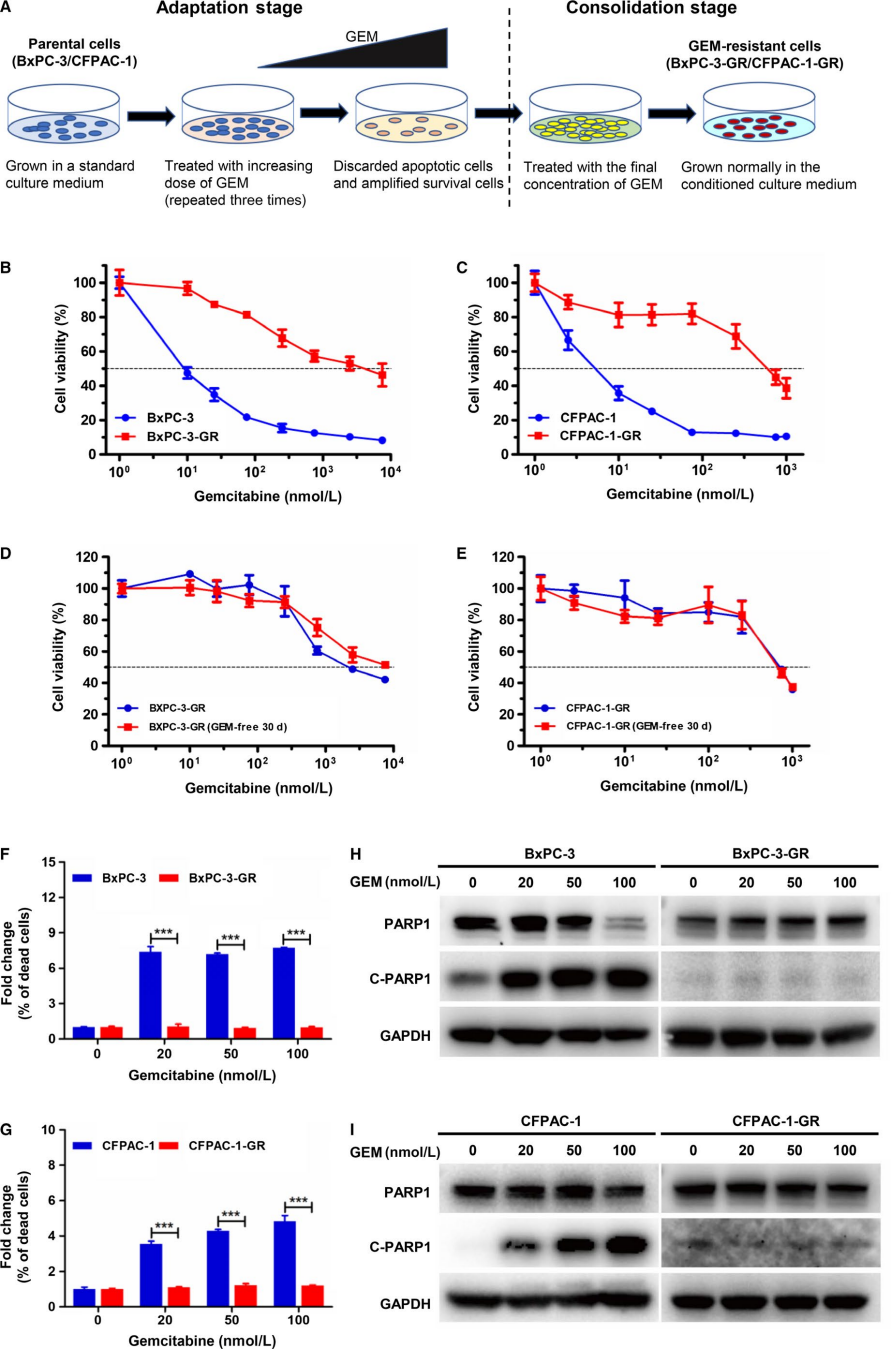
Establishment and characterization of irreversible gemcitabine (GEM) resistance in pancreatic cancer (PC) cell lines. A, Schematic diagram showing the establishment of GEM‐resistant cell lines. Briefly, parental cells were cultured in medium with increasing concentrations of GEM until resistance was acquired to the final concentration. B and C, 3‐(4,5‐Dimethylthiazol‐2‐yl)‐2,5‐diphenyltetrazolium bromide (MTT) assay of parental (BxPC‐3 and CFPAC‐1) and GEM‐resistant (BxPC‐3‐GR and CFPAC‐1‐GR) cells exposed to GEM at different concentrations for 48 h. D and E, GEM sensitivity of BxPC‐3‐GR and CFPAC‐1‐GR cells cultured with GEM‐containing or ‐free medium for 30 d was determined by MTT assays. F and G, Apoptosis rates were significantly lower in GEM‐resistant cells than in their respective parental cell lines at the same GEM dose. H and I, Protein expression of poly adenosine diphosphate ADP‐ribose polymerase 1 (PARP1) and cleaved PARP1 (C‐PARP1) were compared between parental and GEM‐resistant cells using western blot analysis after incubating with different concentrations of GEM for 48 h. Glyceraldehyde 3‐phosphate dehydrogenase (GAPDH) was used as a loading control. ***P < .001; comparisons indicated by lines

Following continuous exposure to GEM in stepwise increments, two GEM‐resistant subgroups BxPC‐3‐GR and CFPAC‐1‐GR were established from their respective parental cell lines (Figure 1A). To assess GEM sensitivity, MTT assays were performed on the BxPC‐3 and CFPAC‐1 parental and GEM‐resistant cell lines. As shown in Figure 1B and C, the effect of GEM inhibition was higher in parental cells than in their respective resistant cells. Gemcitabine IC_50_ values were calculated from the dose‐response curves (Tables 1 and 2). Compared to its parents, the GEM IC_50_ value of BxPC‐3‐GR cells was approximately 111.7‐fold higher (increased from 24 ± 3 to 2680 ± 104 nmol/L), whereas that of CFPAC‐1‐GR cells was more than 210‐fold higher than that of its parents (increased from 3 ± 0.2 to 631 ± 59 nmol/L). Interestingly, both BxPC‐3‐GR and CFPAC‐1‐GR acquired irreversible GEM resistance. Indeed, following culture with GEM‐free medium for 30 days, resistant clones were obstinate when re‐exposed to the drug for 48 hours (Figure 1D,E).

**Table 1:** Drug sensitivity of BxPC‐3 and BxPC‐3‐GR

Drug	BxPC‐3	BxPC‐3‐GR	
	IC_50_ (nmol/L)^a^	IC_50_ (nmol/L)	RI^b^
Gemcitabine	24 ± 3	2680 ± 104	111.7
Docetaxel	0.9 ± 0.5	37 ± 7	41.1
5‐Flourouracil	69 ± 9	2374 ± 210	34.4
Irinotecan	2530 ± 82	40 040 ± 1844	15.8
Capecitabine	7890 ± 911	35 550 ± 10 417	4.5
Oxaliplatin	1606 ± 496	4668 ± 1417	2.9
Cisplatin	1656 ± 284	2373 ± 381	1.4

a The IC_50_ values were defined as the concentration of cells inhibiting growth at 50%.

b Drug resistance index (RI) was determined by dividing IC_50_ values of BxPC‐3 and BxPC‐3‐GR.

**Table 2:** Drug sensitivity of CFPAC‐1 and CFPAC‐1‐GR

Drug	CFPAC‐1	CFPAC‐1‐GR	
	IC_50_ (nmol/L)^a^	IC_50_ (nmol/L)	RI^b^
Gemcitabine	3 ± 0.2	631 ± 59	210.3
Capecitabine	548 ± 20	30 553 ± 830	55.8
5‐Flourouracil	34 ± 3	599 ± 150	17.6
Oxaliplatin	4455 ± 745	16 137 ± 3478	3.6
Irinotecan	1540 ± 792	3437 ± 655	2.2
Docetaxel	30 ± 5	26 ± 9	0.9
Cisplatin	3463 ± 228	1902 ± 162	0.5

a The IC_50_ values were defined as the concentration of cells inhibiting growth at 50%.

b Drug resistance index (RI) was determined by dividing IC_50_ values of CFPAC‐1 and CFPAC‐1‐GR.

To further assess the performance of GEM on parental and GEM‐resistant cells, these cells were incubated in different concentrations of GEM for 48 hours, after which apoptosis was detected using flow cytometry. The percentage of dead cells in each treatment group was normalized to that of untreated samples. As shown in Figure 1F, the apoptotic rate was significantly higher in BxPC‐3 cells than in BxPC‐3‐GR cells at the same dose of GEM. The apoptotic rate of CFPAC‐1 cells was consistently higher at GEM concentrations ranging from 20 to 100 nmol/L, whereas that of CFPAC‐1‐GR cells at the same GEM dose used was significantly lower (Figure 1G). We also examined the apoptosis mechanism in BxPC‐3 and CFPAC‐1 parental and resistant cell lines using immunoblotting analysis. The expression level of cleaved‐PARP1, an apoptosis inducer, increased in BxPC‐3 and CFPAC‐1 cells as GEM concentration increased (Figure 1H,I). However, cleaved‐PARP1 protein expression was lower in BxPC‐3‐GR and CFPAC‐1‐GR cells than in their parental cells at the same GEM dose.

### 3.2 Both GEM‐resistant cells were more resistant to GEM in vivo

**Figure 2:**
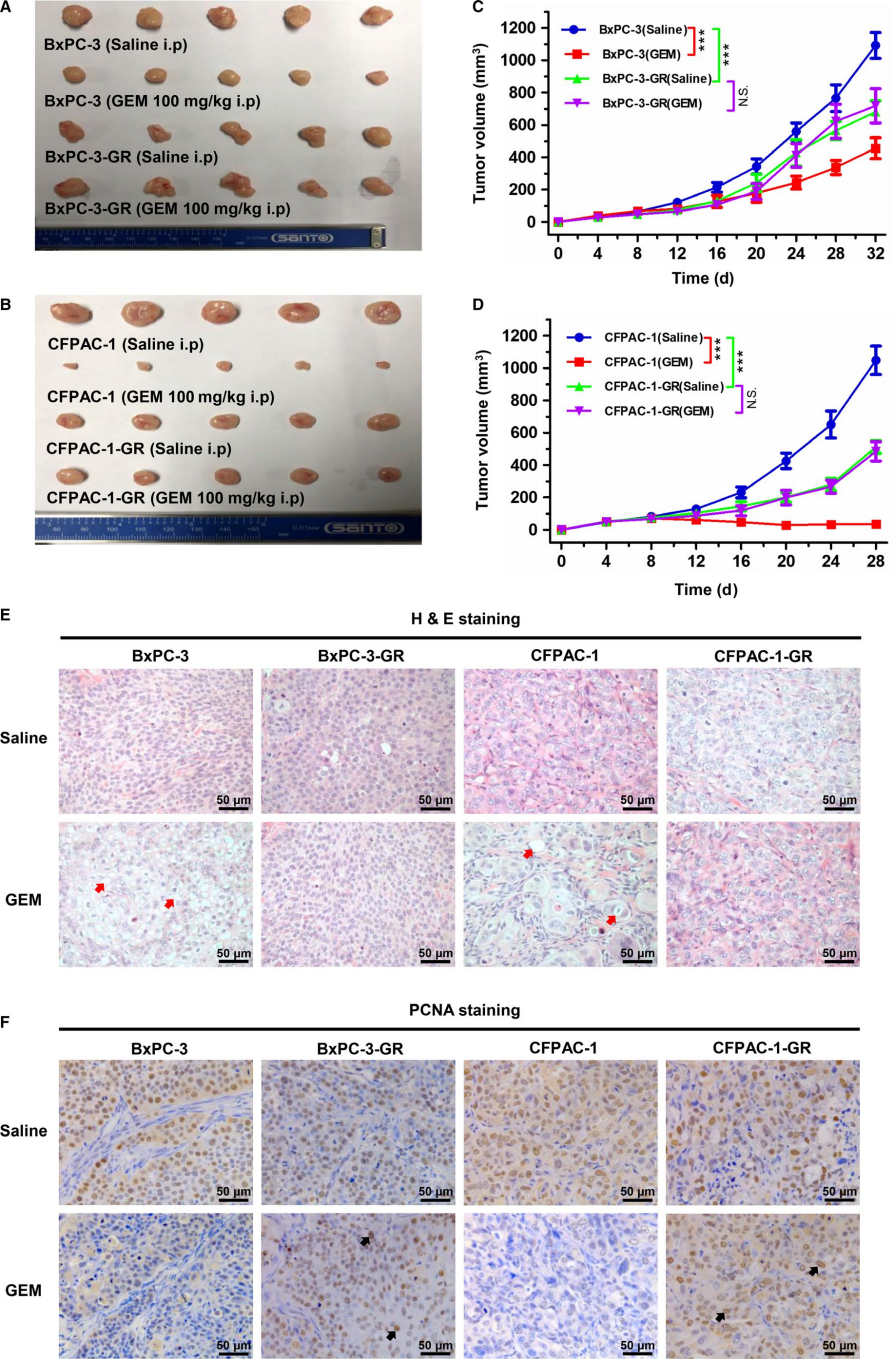
Both gemcitabine (GEM)‐resistant cell lines were more resistant to GEM in vivo. A and B, Tumors were resected from nude mouse model xenografts after different treatments (n = 5). C and D, Tumor growth was significantly inhibited by GEM in parental cells (BxPC‐3 and CFPAC‐1) compared with their corresponding GEM‐resistant cells. E, Representative histological features of BxPC‐3, BxPC‐3‐GR, CFPAC‐1, and CFPAC‐1‐GR tumors following different treatments. Red arrows indicate vacuolization formation and apoptotic features. F, Immunohistochemical staining of proliferation marker proliferating cell nuclear antigen (PCNA) in BxPC‐3, BxPC‐3‐GR, CFPAC‐1, and CFPAC‐1‐GR tumors following different treatments. Black arrows indicate positive cells. ***P < .001; comparisons indicated by lines. i.p, intraperitoneal injection; NS, no significant difference. Scale bar, 50 µm

To investigate drug resistance in vivo, both GEM‐resistant and parental cells were subcutaneously injected in mice. As shown in Figure 2A‐D, GEM greatly inhibited tumor size and growth curves in BxPC‐3 and CFPAC‐1 cells, whereas no significant effect on GEM‐resistant cells was observed. Furthermore, the proliferation ability of BxPC‐3‐GR and CFPAC‐1‐GR cells was significantly reduced in vivo compared to their respective parental cells. Subsequently, tumor tissue sections were prepared and stained with H & E and PCNA. Compared to BxPC‐3 and CFPAC‐1 cells, fewer BxPC‐3‐GR and CFPAC‐1‐GR cells displayed vacuolization formation and apoptotic features with GEM treatment (Figure 2E), but more GEM‐resistant cells were PCNA positive (Figure 2F), demonstrating their resistance to GEM in vivo.

### 3.3 Morphological and biological characterization of GEM‐resistant cell lines

**Figure 3:**
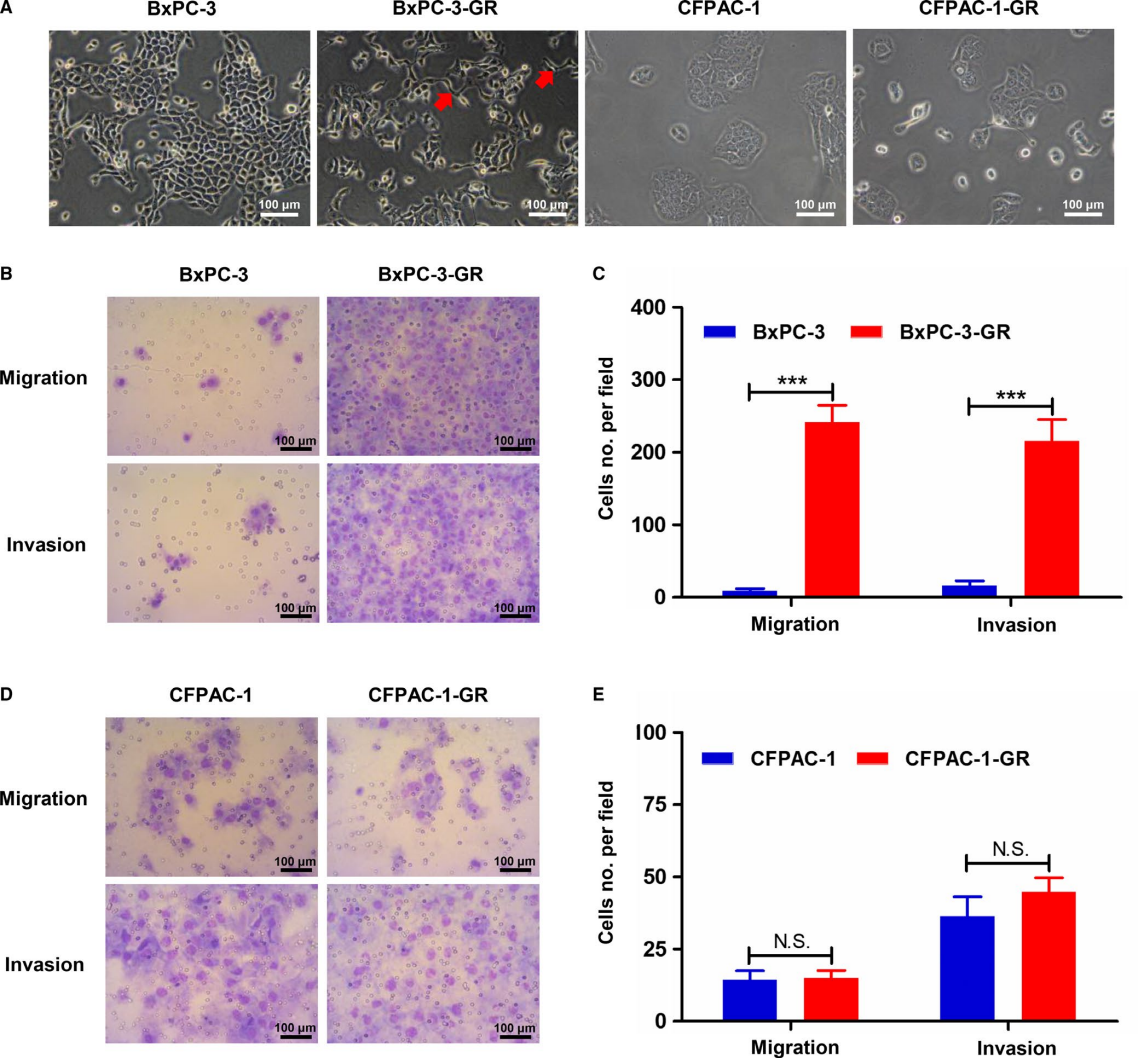
Morphological and metastatic characteristics of parental and gemcitabine (GEM)‐resistant cell lines. A, Morphological characteristics of parental and GEM‐resistant cells were determined using an inverted microscope. Red arrows indicate cellular pseudopods. B and C, Transwell assays showed increased migratory and invasive abilities among BxPC‐3‐GR cells compared with BxPC‐3 cells. D and E, Comparisons of migration and invasion between CFPAC‐1 and CFPAC‐1‐GR cells using transwell assays detected no significant differences. ***P < .001; comparisons indicated by lines. NS, no significant difference. Scale bar, 100 µm

Morphological and biological changes are generally thought to come along with drug resistance.^20^ As shown in Figure 3A, BxPC‐3‐GR cells lost cell‐cell adhesion and, increasingly, exhibited the mesenchymal phenotype, compared with parental cells. However, there was no morphological difference between CFPAC‐1 and CFPAC‐1‐GR. In this context, we further analyzed the migratory and invasive ability of these cells using transwell chambers and Matrigel invasion assays, respectively. BxPC‐3‐GR consistently showed a significant increase in migratory and invasive ability (Figure 3B,C), whereas no significant change in metastasis and invasion capacity was observed between CFPAC‐1 and CFAPC‐1‐GR (Figure 3D,E).

**Figure 4:**
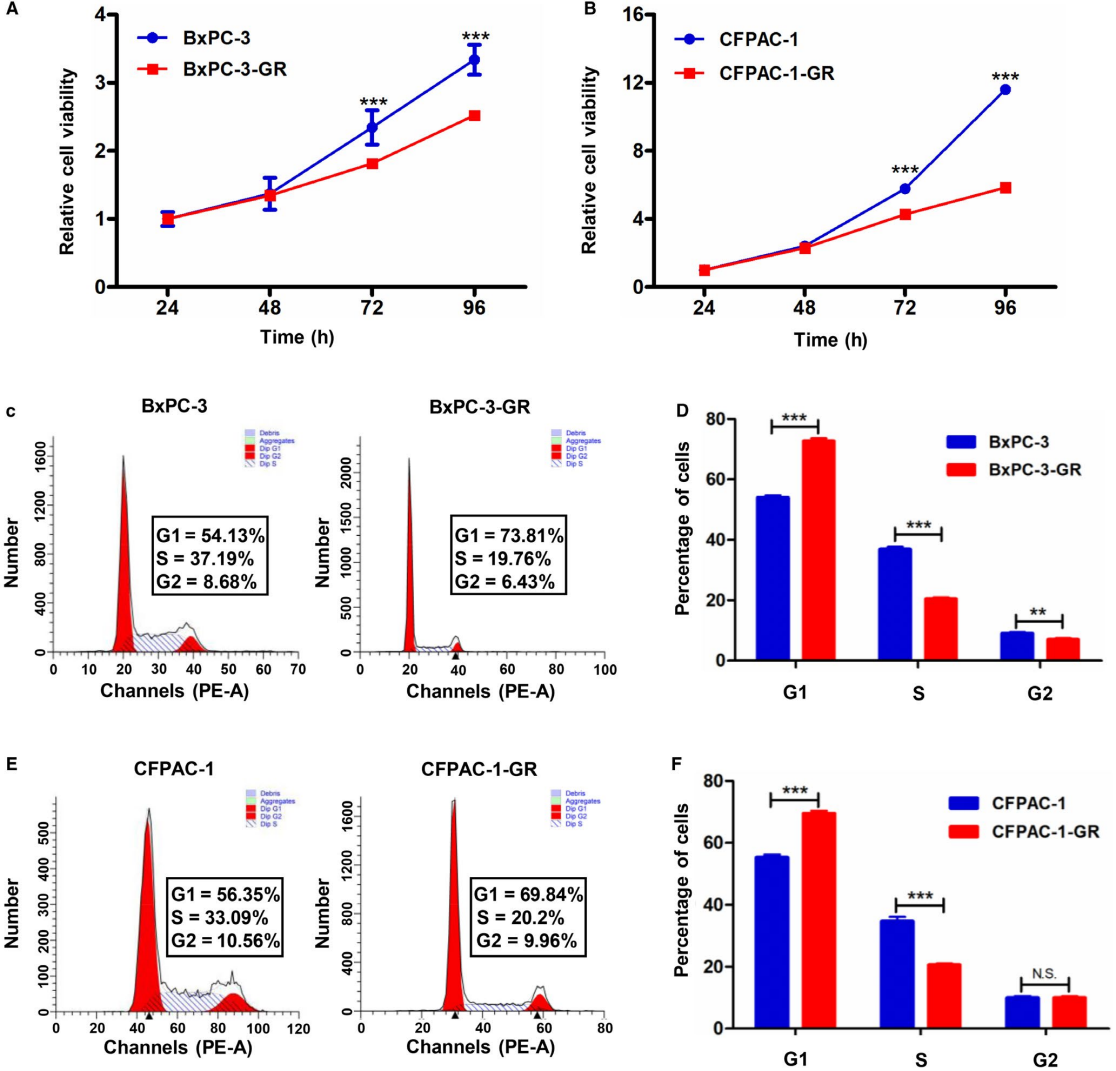
Proliferative capacity and cell cycle distribution in gemcitabine (GEM)‐resistant and parental cell lines. A and B, CCK8 assays showed inhibited proliferation in GEM‐resistant cells (BxPC‐3‐GR and CFPAC1‐GR) compared with their corresponding parental cells. C, Representative images of cell cycle distribution analysis in BxPC‐3 and BxPC‐3‐GR cells. D, Quantification of cell cycle distribution analysis revealed that BxPC‐3‐GR cells were arrested in the G1 phase. E, Representative images of cell cycle distribution analysis in CFPAC‐1 and CFPAC‐1‐GR cells. F, Quantification of cell cycle distribution analysis revealed that CFPAC‐1‐GR cells were arrested in the G1 phase. **P < .01, ***P < .001; comparisons indicated by lines. NS, no significant difference

To compare the proliferation ability between GEM‐resistant and parental cells, we performed CCK8 assays. Proliferation was significantly lower in BxPC‐3‐GR cells than in parental cells (Figure 4A); similar results were observed in CFAPC‐1‐GR (Figure 4B). We then determined cell cycle distributions in GEM‐resistant and parental cells using flow cytometry. As shown in Figure 4C and D, significantly fewer cells were observed in the S and G2 phases among GEM‐resistant BxPC‐3‐GR cells (20.41 ± 0.57% and 6.96 ± 0.46%, respectively) than among parental cells (36.84 ± 0.86 and 9.11 ± 0.42%, respectively). The proportion of CFPAC‐1‐GR cells (20.54 ± 0.5%) in the S phase was consistently significantly lower than that of CFPAC‐1 cells (34.71 ± 1.41%) (Figure 4E,F). However, proportions of cells in the G1 phase among BxPC‐3‐GR and CFPAC‐1‐GR (72.63 ± 1.02% and 69.49 ± 0.89%, respectively) were significantly higher than those of BxPC‐3 and CFPAC‐1 cells (54.05 ± 0.62% and 55.31 ± 0.91%, respectively) (Figure 4D,F).

### 3.4 Different drug‐resistant profiles in both GEM‐resistant cell lines

To further explore the drug‐resistant characteristics of BxPC‐3‐GR and CFPAC‐1‐GR, we evaluated their sensitivity to several different chemotherapeutic agents that are commonly used in clinical settings; the results are summarized in Tables 1 and 2. 3‐(4,5‐dimethylthiazol‐2‐yl)‐2,5‐diphenyltetrazolium bromide assays showed that GEM‐resistant cell lines had higher resistance than parental cell lines to 5‐flourouracil, capecitabine, irinotecan, and oxaliplatin. Notably, BxPC‐3‐GR showed higher resistance to docetaxel (41.4‐fold) and cisplatin (1.4‐fold), whereas CFPAC‐1‐GR became more sensitive. These findings suggest that both BxPC‐3‐GR and CFPAC‐1‐GR are multiple‐drug resistant cell lines, but with different drug‐resistant profiles.

### 3.5 Overview of mRNA expression and enrichment analyses between GEM‐resistant and parental cell lines

**Figure 5:**
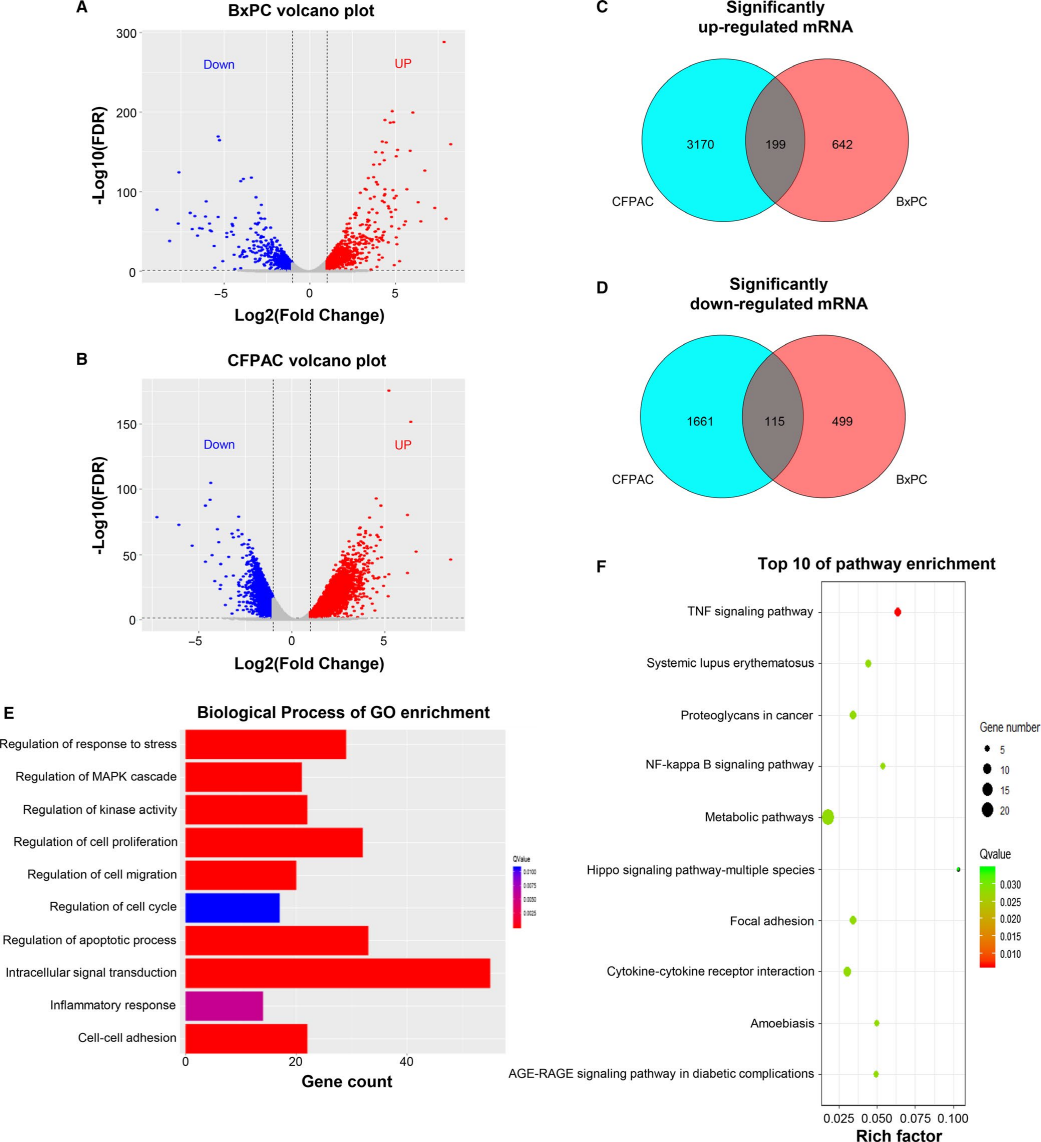
Overview of mRNA expression and enrichment analyses between gemcitabine (GEM)‐resistant and parental cell lines. A and B, Volcano figure showing significantly differentially expressed (SDE) genes in BxPC‐3‐GR and CFPAC‐1‐GR cells compared to their respective parental cells. Red and blue dots indicate significantly up‐ and downregulated genes in GEM‐resistant cells, respectively. C and D, Venn diagrams show consistently up‐ and downregulated mRNAs in the GEM‐resistant cell lines. E, Biological processes identified by gene ontology (GO) enrichment analysis based on consistent SDE genes. F, Top 10 results of Kyoto Encyclopedia of Genes and Genomes pathway enrichment analysis based on consistent SDE genes. FDR, false discovery rate

Given different phenotypes between GEM‐resistant and parental cells, we next analyzed mRNA profiles among these cells using RNA sequencing. Compared to their respective parental cells, we identified 1455 and 5145 significantly differentially expressed (SDE) mRNAs in the BxPC‐3‐GR and CFPAC‐1‐GR cells (Figure 5A,B). In BxPC‐3‐GR cells, 841 mRNAs were significantly upregulated and 614 mRNAs were significantly downregulated. In CFPAC‐1‐GR, 3369 mRNAs were significantly upregulated and 1776 mRNAs were significantly downregulated. To explore common gene expression phenomena among drug‐resistant cell lines, we analyzed significantly up‐ and downregulated mRNAs among the BxPC‐3‐GR and CFPAC‐1‐GR cells and found 199 mRNAs that were consistently upregulated and 115 that were consistently downregulated in both cell lines (Figure 5C,D). We also performed GO and KEGG pathway enrichment analyses on SDE mRNAs that were consistently regulated in both GEM‐resistant cell lines. Significantly different biological processes between the two groups included regulation of apoptotic process, cell‐cell adhesion, and regulation of cell cycle, which may be related to chemoresistance (Figure 5E). As shown in Figure 5F, the top 10 signaling pathways with a *Q* value <0.05 were significantly enriched. The most significantly enriched pathway was the tumor necrosis factor signaling pathway.

### 3.6 Expression of RRM1, STIM1, and TRIM21 was associated with the level of the acquired GEM resistance

**Figure 6:**
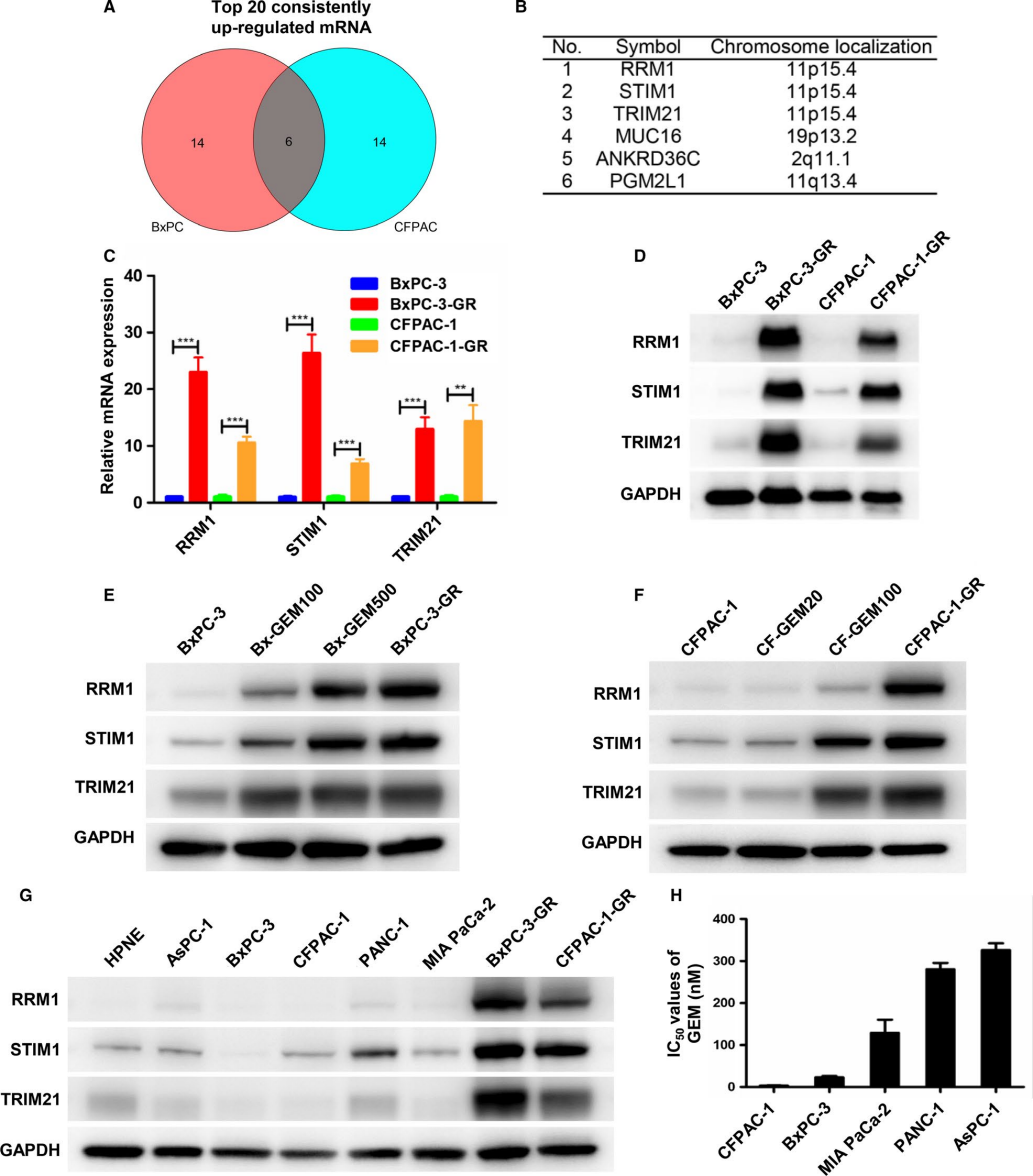
Expression of RRM1, STIM1, and TRIM21 was associated with the level of acquired gemcitabine (GEM) resistance. A, Venn diagram showed the top 20 consistently upregulated mRNAs in both GEM‐resistant cell lines. B, Six intersected genes selected from among the top 20 consistently upregulated mRNAs. C and D, Total RNA and cytoplasmic proteins were extracted from BxPC‐3, BxPC‐3‐GR, CFPAC‐1, and CFPAC‐1‐GR. mRNA and protein expression levels of RRM1, STIM1, and TRIM21 were determined by quantitative reverse‐transcription polymerase chain reaction and western blot analysis. Glyceraldehyde 3‐phosphate dehydrogenase (GAPDH) was used as a control. E and F, RRM1, STIM1, and TRIM21 protein expression levels were measured by western blot analysis in both parental cell lines and their derived GEM subclones. GAPDH was used as a control. G, RRM1, STIM1, and TRIM21 protein expression levels among normal pancreatic and pancreatic cancer (PC) cell lines were evaluated by western blot analysis. GAPDH was used as a control. H, Histogram shows IC_50_ values for GEM in AsPC‐1, BxPC‐3, CFPAC‐1, MIA PaCa‐2, and PANC‐1 cell lines. **P < .01, ***P < .001; comparisons indicated by lines

To further explore the molecular mechanisms underlying GEM resistance acquisition, we analyzed the top 20 consistently upregulated mRNAs in both GEM‐resistant cell lines (Figure 6A; Tables S1 and S2). Six intersected genes (RRM1, STIM1, TRIM21, MUC16, ANKRD36C, and PGM2L1) are shown in Figure 6B. RRM1, STIM1, and TRIM21 were picked out for their potential relation to drug resistance.^21^, ^22^, ^23^, ^24^ As shown in Figure 6C and D, mRNA and protein expression levels of RRM1, STIM1, and TRIM21 were significantly higher in BxPC‐3‐GR and CFPAC‐1‐GR cells than in their corresponding parental cells. We then estimated RRM1, STIM1, and TRIM21 protein expression among different Bx‐GEM subclones with differential grades of resistance to 100 (Bx‐GEM100), 500 (Bx‐GEM500), and 1000 nmol/L (BxPC‐3‐GR) GEM (Figure 6E). Protein expression level was directly related to the grade of GEM resistance; a similar result was observed among the different CF‐GEM subclones (Figure 6F). To further validate this conclusion, basal expression of RRM1, STIM1, and TRIM21 was detected in pancreatic normal and cancer cell lines using western blot analysis. As shown in Figure 6G, expression levels of RRM1, STIM1, and TRIM21 were low in BxPC‐3 and CFPAC‐1 cells, higher in PANC‐1 cells, and highest among both GEM‐resistant cells, which was consistent with the degree of GEM resistance detected in PC cell lines by MTT assays (Figure 6H). Together, these findings suggest that RRM1, STIM1, and TRIM21 expression levels are directly correlated to GEM resistance levels.

### 3.7 Abnormal expression and prognostic role of RRM1, STIM1, and TRIM21 in PC

**Figure 7:**
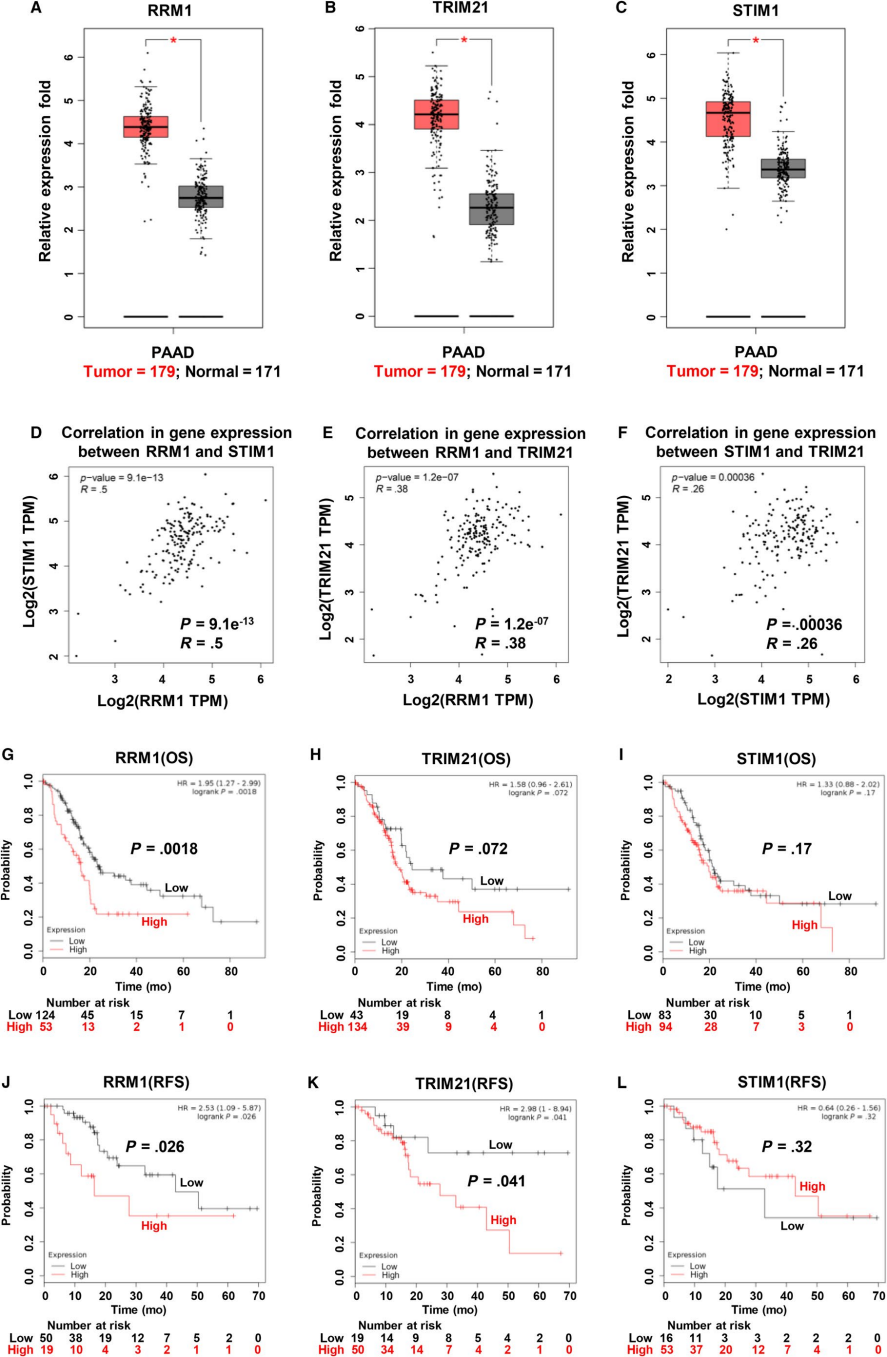
Abnormal expression and prognostic role of RRM1, STIM1, and TRIM21 in pancreatic cancer (PC). A–C, RRM1, TRIM21, and STIM1 mRNA expression levels were significantly higher in PC tissues than in corresponding adjacent normal tissues based on TCGA database search results. Association of (D) mRNA expression with RRM1 and STIM1, (E) mRNA expression with RRM1 and TRIM21, and (F) mRNA expression with STIM1 and TRIM21 assessed using Spearman's correlation based on GEPIA online database. G–I, Overall survival (OS) curves for RRM1, TRIM21, and STIM1 in PC patients, plotted using the Kaplan‐Meier method. J–L, Recurrence‐free survival (RFS) curves for RRM1, TRIM21, and STIM1 in PC patients, plotted using the Kaplan‐Meier method. *P < .05; comparisons indicated by lines. PAAD, pancreatic adenocarcinoma

Based on these results, we first assessed the expression of these three genes in PC and corresponding normal tissues using a TCGA pan‐cancer dataset obtained from the GEPIA online database. As shown in Figure 7A‐C, RRM1, TRIM21, and STIM1 were more highly expressed in PC tissues than in corresponding normal tissues (*P* < .05). Interestingly, pair‐wise gene correlation analysis based on the TCGA database showed a significantly positive correlation of mRNA expression between RRM1 and STIM1 in PC tissues (Figure 7D). Similar results were shown between RRM1 and TRIM21 (Figure 7E), and between STIM1 and TRIM21 (Figure 7F). To evaluate prognostic values of these three potential mRNAs in PC patients, the KM plotter database was utilized. As shown in Figure 7G and J, there was a significant decrease in overall survival (OS, *P* = .0018) and recurrence‐free survival (RFS; *P* = .026) in the RRM1 high cohort compared with the low cohort. Although the difference in OS was not significantly different between the TRIM21 high and low groups (*P* = .072) (Figure 7H), low TRIM21 expression was significantly correlated with increased RFS (*P* = .041) (Figure 7K). There was no significant difference in OS (*P* = .17) and RFS (*P* = .32) between the STIM1 high and low cohorts (Figure 7I,L).

## 4 DISCUSSION

In this study, we investigated acquired chemoresistance in PC using chemo‐resistant cell models. Through continuous exposure to GEM using a dose escalation strategy, two stable GEM‐resistant cell subgroups (BxPC‐3‐GR and CFAC‐1‐GR) were established from parental cell lines. Unlike previously described drug‐resistant cell models,^25^, ^26^ acquired GEM resistance was an irreversible phenomenon in both the BxPC‐3‐GR and CFPAC‐1‐GR cell lines, making these lines more suitable for subsequent analyses of drug resistance.

Changes in the morphological and biological characteristics of resistant cells can denote acquired drug resistance. Funamizu et al^27^ reported that two resistant cell lines exhibited increased cell growth, as compared to its parents. In contrast, both of our GEM‐resistant cell lines showed a significant decrease in proliferative capability in vitro and in vivo. These differences could be due to cell cycle disturbance, since the cell cycle of GEM‐resistant cells was arrested at the G1 phase compared with parental cells, providing cancer cells with sufficient time to respond to DNA damage.^28^ Several previous studies have reported that cancer metastasis and drug resistance are closely related.^20^, ^29^ In the present study, BxPC‐3‐GR cells exhibited a mesenchymal phenotype with enhanced migration and invasion. However, there was no change in morphology or motility in CFPA‐1‐GR cells compared with parental CFPAC‐1 cells. We hypothesized that the different biological characteristics of the two GEM‐resistant cell lines may have been caused by different final concentrations of the drug.

Several recent retrospective studies and clinical trials have been performed to compare the efficacy of GEM alone or combined with other chemotherapeutic agents; however, combined regimens such as erlotinib, nab‐paclitaxel, and oxaliplatin have not markedly improved OS results of patients with PC.^30^, ^31^, ^32^, ^33^ One of the main causes of poor efficacy of combination chemotherapy is cross‐resistance to multiple drugs. In our study, both BxPC‐3‐GR and CFPAC‐1‐GR were cross‐resistant to 5‐flourouracil, capecitabine, irinotecan, and oxaliplatin. Interestingly, BxPC‐3‐GR showed additional resistance to docetaxel and cisplatin, whereas CFPAC‐1‐GR became more sensitive. These findings suggest that both BxPC‐3‐GR and CFPAC‐1‐GR are multiple‐drug resistant cell lines, but with different drug‐resistant profiles.

Many important signaling pathways and molecular targets have been confirmed to be correlated with chemotherapy resistance in PC. Irreversible therapeutic resistance remains a challenge for patients receiving chemotherapy. Therefore, we further explored potential targets for regulation of GEM‐acquired resistance through transcriptome sequencing.

Previous studies have demonstrated that RRM1, an enzyme indispensable for drug metabolism regulation, plays an important role in chemotherapy resistance in multiple types of cancers.^22^, ^34^, ^35^, ^36^ Consistently, our quantitative reverse‐transcription polymerase chain reaction and western blot analysis illustrated that RRM1 mRNA and protein expression were significantly upregulated in both GEM‐resistant cell lines. We also demonstrated that RRM1 expression in PC cell lines and different GEM‐resistant subclones was correlated with the grade of GEM resistance. In addition to RRM1, we identified two genes (STIM1 and TRIM21) related to acquired GEM resistance. STIM1, a major component of store‐operated calcium channels in regulating Ca^2+^ influx, has been reported to take part in several physiological and pathological processes.^37^, ^38^ Knockdown of STIM1 expression has been shown to increase the chemosensitivity of 5‐fluorouracil or GEM in pancreatic adenocarcinoma cell lines.^23^ However, Gualdani et al^39^ showed that knockdown of STIM1 expression dramatically reduced cisplatin cytotoxicity in non‐small‐cell lung carcinoma cells by inhibiting the DNA damage repair pathway. These previous findings suggest that STIM1 may have a dual role in regulating chemoresistance in different types of cancers. TRIM21 has been shown to decrease the response to cisplatin in colon cancer and PC by downregulating Par‐4 expression, whereas high‐level TRIM21 expression was correlated with poor OS in PC patients.^24^ Based on these findings, we further demonstrated that these three genes were more highly expressed in PC tissues than in corresponding normal tissues and that high RRM1 and TRIM21 expression indicated poor prognosis in PC patients. However, further tissue samples are needed to sufficiently demonstrate and validate this conclusion and the detailed molecular mechanisms by which these three genes regulate acquired chemoresistance in PC.

In conclusion, we successfully established two stable GEM‐resistant cell subclones and determined that RRM1, STIM1, and TRIM21 are potential biomarkers for response to GEM in patients with PC.

## CONFLICT OF INTEREST

None declared.

## AUTHORS' CONTRIBUTIONS

WLW, JRZ, and LSZ designed the study. JRZ and LSZ participated in majority of the experiments and wrote the manuscript. HLZ, WHG, and YH conducted the animal experiments. YCY and XHZ performed the statistical analysis of experimental data. WZ and YK analyzed the transcriptome sequencing data. YD reviewed and revised the manuscript. All authors agreed to submit the final manuscript.

## Data Availability

The data that support the findings of this study are available from the corresponding author upon reasonable request.
