# Supplementary material for: Identification of chemoresistance‐related mRNAs based on gemcitabine‐resistant pancreatic cancer cell lines
Source: Cancer Med. 2019 Dec 11;9(3):1115–30. doi: 10.1002/cam4.2764 (PMC6997050; doi:10.1002/cam4.2764)
Supplement: Supplementary file 1 [file CAM4-9-1115-s001.docx]

| Table S1. Top 20 consistently up-regulated mRNA in BxPC-3-GR compared to BxPC-3. | | | | |
| --- | --- | --- | --- | --- |
| No. | Symbol | Description | Log2FC | Q value |
| 1 | MUC16 | Mucin 16, cell surface associated | 7.84 | 7.80E-289 |
| 2 | MUC4 | Mucin 4, cell surface associated | 4.93 | 6.52E-93 |
| 3 | TRIM68 | Tripartite motif containing 68 | 4.69 | 1.33E-187 |
| 4 | MCAM | Melanoma cell adhesion molecule | 4.61 | 4.36E-80 |
| 5 | STIM1 | Stromal interaction molecule 1 | 4.48 | 6.40E-163 |
| 6 | TRIM21 | Tripartite motif containing 21 | 4.40 | 1.81E-190 |
| 7 | TRIM22 | Tripartite motif containing 22 | 4.40 | 7.77E-104 |
| 8 | TRIM34 | Tripartite motif containing 34 | 4.35 | 2.75E-96 |
| 9 | IL1R1 | Interleukin 1 receptor type 1 | 4.25 | 4.33E-89 |
| 10 | TRIM5 | Tripartite motif containing 5 | 4.06 | 2.41E-135 |
| 11 | RRM1 | Ribonucleotide reductase catalytic subunit M1 | 3.83 | 3.17E-150 |
| 12 | TIMM10B | Translocase of inner mitochondrial membrane 10B | 3.72 | 1.03E-134 |
| 13 | PRKAR2B | Protein kinase cAMP-dependent type II regulatory subunit beta | 3.45 | 2.69E-24 |
| 14 | ANKRD36C | Ankyrin repeat domain 36C | 3.43 | 5.31E-56 |
| 15 | AGO4 | Argonaute 4, RISC catalytic component | 3.40 | 1.08E-65 |
| 16 | RGL1 | Ral guanine nucleotide dissociation stimulator like 1 | 3.24 | 2.39E-44 |
| 17 | PGM2L1 | Phosphoglucomutase 2 like 1 | 3.09 | 4.45E-44 |
| 18 | TNFSF18 | TNF superfamily member 18 | 3.05 | 1.68E-16 |
| 19 | CXCL8 | C-X-C motif chemokine ligand 8 | 2.86 | 2.58E-80 |
| 20 | MAN1A1 | Mannosidase alpha class 1A member 1 | 2.84 | 1.89E-23 |

Abbreviations: FC means fold change; The Q value refers to the P value after multiple corrections.
