# Supplementary material for: Identification of chemoresistance‐related mRNAs based on gemcitabine‐resistant pancreatic cancer cell lines
Source: Cancer Med. 2019 Dec 11;9(3):1115–30. doi: 10.1002/cam4.2764 (PMC6997050; doi:10.1002/cam4.2764)
Supplement: Supplementary file 2 [file CAM4-9-1115-s002.docx]

| Table S2. Top 20 consistently up-regulated mRNA in CFPAC-1-GR compared to CFPAC-1. | | | | |
| --- | --- | --- | --- | --- |
| No. | Symbol | Description | Log2FC | Q value |
| 1 | RRM1 | Ribonucleotide reductase catalytic subunit M1 | 5.24 | 2.03E-176 |
| 2 | ANKRD36C | Ankyrin repeat domain 36C | 4.45 | 1.65E-32 |
| 3 | RB1CC1 | RB1 inducible coiled-coil 1 | 3.83 | 1.23E-47 |
| 4 | IKZF2 | IKAROS family zinc finger 2 | 3.60 | 1.61E-27 |
| 5 | ITGB8 | Integrin subunit beta 8 | 3.57 | 1.40E-59 |
| 6 | MUC16 | Mucin 16, cell surface associated | 3.55 | 2.14E-25 |
| 7 | KLHL24 | Kelch like family member 24 | 3.52 | 4.41E-24 |
| 8 | PGM2L1 | Phosphoglucomutase 2 like 1 | 3.50 | 8.29E-31 |
| 9 | DMXL2 | Dmx like 2 | 3.48 | 2.75E-37 |
| 10 | BIRC3 | Baculoviral IAP repeat containing 3 | 3.48 | 3.66E-22 |
| 11 | ZNF711 | Zinc finger protein 711 | 3.43 | 1.04E-17 |
| 12 | ANKRD36B | Ankyrin repeat domain 36B | 3.43 | 9.88E-16 |
| 13 | JMY | Junction mediating and regulatory protein, p53 cofactor | 3.28 | 2.11E-28 |
| 14 | ZC3H11A | Zinc finger CCCH-type containing 11A | 3.27 | 1.71E-21 |
| 15 | LRRC8D | Leucine rich repeat containing 8 VRAC subunit D | 3.24 | 1.15E-27 |
| 16 | RNF19A | Ring finger protein 19A, RBR E3 ubiquitin protein ligase | 3.11 | 6.19E-40 |
| 17 | TRIM21 | Tripartite motif containing 21 | 3.09 | 5.10E-61 |
| 18 | STIM1 | Stromal interaction molecule 1 | 3.05 | 5.07E-27 |
| 19 | RALGPS2 | Ral GEF with PH domain and SH3 binding motif 2 | 3.02 | 3.13E-24 |
| 20 | LRRCC1 | Leucine rich repeat and coiled-coil centrosomal protein 1 | 3.02 | 2.34E-21 |

Abbreviations: FC means fold change; The Q value refers to the P value after multiple corrections.
